# A Fiber‐Based 3D Lithium Host for Lean Electrolyte Lithium Metal Batteries

**DOI:** 10.1002/advs.202104829

**Published:** 2022-02-01

**Authors:** Sicen Yu, Zhaohui Wu, John Holoubek, Haodong Liu, Emma Hopkins, Yuxuan Xiao, Xing Xing, Myeong Hwan Lee, Ping Liu

**Affiliations:** ^1^ Program of Materials Science University of California San Diego La Jolla CA 92093 USA; ^2^ Department of NanoEngineering University of California San Diego La Jolla CA 92093 USA; ^3^ Program of Chemical Engineering University of California San Diego La Jolla CA 92093 USA

**Keywords:** 3D host, high porosity, lithium metal anode, RbNO_3_, vapor‐grown carbon fiber

## Abstract

3D hosts are promising to extend the cycle life of lithium metal anodes but have rarely been implemented with lean electrolytes thus impacting the practical cell energy density. To overcome this challenge, a 3D host that is lightweight and easy to fabricate with optimum pore size that enables full utilization of its pore volume, essential for lean electrolyte operations, is reported. The host is fabricated by casting a VGCF (vapor‐grown carbon fiber)‐based slurry loaded with a sparingly soluble rubidium nitrate salt as an additive. The network of fibers generates uniform pores of ≈3 µm in diameter with a porosity of 80%, while the nitrate additive enhances lithiophilicity. This 3D host delivers an average coulombic efficiency of 99.36% at 1 mA cm^−2^ and 1 mAh cm^−2^ for over 860 cycles in half‐cell tests. Full cells containing an anode with 1.35‐fold excess lithium paired with LiNi_0.8_Mn_0.1_Co_0.1_O_2_ (NMC811) cathodes exhibit capacity retention of 80% over 176 cycles at C/2 under a lean electrolyte condition of 3 g Ah^−1^. This work provides a facile and scalable method to advance 3D lithium hosts closer to practical lithium‐metal batteries.

## Graphical abstract

A high‐porosity vapor‐grown carbon fiber (VGCF) framework with embedded RbNO_3_ provides a lithium metal host with a volumetric specific capacity of 1643 mAh cm^−3^ while offering a cumulative capacity of 260.6 Ah cm^−3^ in half‐cell tests. It is shown that host with the optimum combination of pore size and lithiophilicity is essential to ensure full utilization of the porosity.

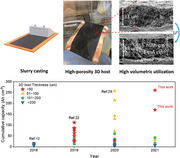

## 1 Introduction

The drive to reduce the cost of electric vehicles has motivated the development of lithium‐metal batteries (LMBs) that promise a specific energy of 500 Wh kg^−1^.^[^ ^1^, ^2^, ^3^ ^]^ Lithium metal is an ideal anode owing to its high capacity of 3860 mAh g^−1^/2046 mAh cm^−3^ and its low reduction potential.^[^ ^4^ ^]^ However, the commercialization of LMBs is hindered by their poor cycle life, which is primarily determined by the plating‐stripping behavior of the lithium metal anode, including the formation of lithium dendrites, large volume change, and the continuous formation of dead lithium during cycling.^[^ ^5^ ^]^ Among reported approaches, 3D hosts are expected to accommodate volume changes during Li plating/stripping and enable stable cycling performance originating from their low and homogeneous effective current density.^[^ ^5^, ^6^, ^7^ ^]^

The ideal 3D host should restrict Li deposition to within its pores, avoiding any deposition on the outside. To achieve this, the host needs to have low‐tortuosity and the proper pore size. It has been shown that the pore size distribution of the 3D host has a significant impact on the Li morphology.^[^ ^8^ ^]^ For example, Cu structures with an average pore size of 5 µm exhibit more compact and uniform Li deposition than larger or smaller average pore sizes. Low electrode tortuosity can mitigate the uneven ion concentration gradient inside the porous electrode and reduce local current density on the upper surface.^[^ ^9^ ^]^ Further, the host needs to be highly lithiophilic.^[^ ^7^, ^9^, ^10^, ^11^, ^12^, ^13^ ^]^ Introducing electronegative sites as Lewis bases have been found to induce stronger interactions with Lewis acidic lithium ions to achieve uniform lithium nucleation.^[^ ^14^ ^]^ Modifying the surface of the 3D host by grafting functional groups or coating it with a lithiophilic layer is also widely employed to achieve this effect.^[^ ^15^, ^16^ ^]^ Additionally, introducing nitrate additives is highly effective in optimizing lithium morphology in both ether‐based and carbonate‐based electrolytes.^[^ ^17^, ^18^, ^19^, ^20^, ^21^ ^]^ Embedding LiNO_3_ in a carbon‐based 3D host has been shown to improve lithium morphology inside the 3D host due to optimized solid electrolyte interphase (SEI) compositions that contain Li_3_N, LiN*_x_* O*_y_*, and a large amount of LiF.^[^ ^22^ ^]^ LiNO_3_ is sparingly soluble in carbonates. However, the solid LiNO_3_ in the 3D host structure serves as a reservoir to provide a continuous source of nitrate ions to help regulate lithium plating.

Further development of the structure and the fabrication processes for the 3D hosts are still needed for practical applications. Various processes including electrospinning, freezing drying, de‐alloying, and lithography have been employed, but a process that can utilize the current battery manufacturing process would be highly desirable.^[^ ^6^, ^9^ ^]^ The 3D structure needs to be readily built on a current collector to be welded with tabs easily. Perhaps most importantly, the 3D hosts should be made of lightweight materials with high porosities, and the amount of lithiophilic agents, including nitrates, should be as low as possible to reduce inactive weight and volume. The pores should also be utilized as much as possible to translate these electrode performances into high energy density at the cell level.^[^ ^23^ ^]^ In this regard, carbon‐based hosts are more desirable than metal‐based hosts.^[^ ^5^ ^]^ Recent analysis shows that the anode specific and volumetric capacities should be more than 1100 mAh g^−1^ and 841 mAh cm^−3^, respectively, to show a decisive advantage over graphite, tin, and metal oxide‐based anode materials.^[^ ^24^ ^]^

**Figure 1:**
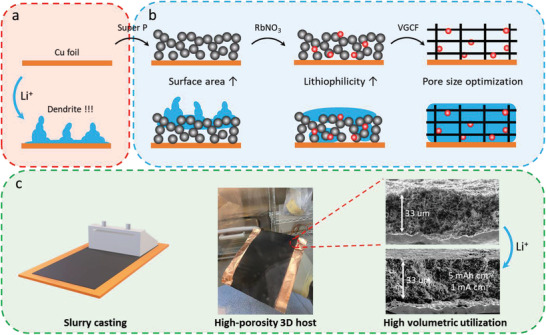
Schematic diagram of 3D hosts for Li metal delineating the effect of pore size and lithiophilicity offered by RbNO_3_. a) Cu foil where Li deposits as Li dendrites; b) the design guideline for 3D host where high loading Li deposits inside the host. A host made of Super P only is not lithiophilic enough. Adding nitrates will enhance lithiophilicity but the host pore sizes are too small. VGCF decorated with nitrates provides optimum combination of pore size and lithiophilicity; and c) the fabrication of high‐porosity 3D host with nitrates and large pore size by slurry casting. A 33‐µm thick coating houses 5 mAh cm^−2^ of lithium, close to its theoretical capacity.

In this work, we report high‐performance 3D hosts composed of composites of carbon materials and RbNO_3_ (**Figure** **1**). The added nitrate makes the host lithiophilic, which enables us to investigate carbon materials with different particle shapes and sizes to control the hosts' pore sizes. We have found that even with ensured lithiophilicity, the electrode pore size is critical to encourage lithium deposition inside the host. Vapor‐grown carbon fiber (VGCF) has been identified to form a host through a slurry‐casting method with an average pore size of 3 µm and porosity as high as 80%. RbNO_3,_ as compared to previously reported LiNO_3,_ has even lower solubility in carbonate electrolytes. As a result, a low loading will still enable the gradual release of nitrate to enable dendrite‐free lithium plating. This host provides a volumetric specific capacity of 1643 mAh cm^−3^ while offering a cumulative capacity of 260.6 Ah cm^−3^ in half‐cell tests before cell failure. This record‐setting performance is utilized in a full cell with and LiNi_0.8_Mn_0.1_Co_0.1_O_2_ (NMC811) cathode. Our findings are essential for future high‐capacity 3D host design by clearly delineating the roles of pore size and host lithiophilicity.

## 2 Results and Discussion

### 2.1 Design of 3D Hosts

The design guideline for 3D hosts is illustrated in Figure 1b to delineate the respective roles of host lithiophilicity and pore size. In the absence of a nitrate salt as a lithiophilic agent, lithium preferentially deposits outside of the host as carbon‐based hosts are not sufficiently lithiophilic. With the addition of the nitrate lithiophilic agent, lithium will first deposit inside the host with desired morphology when the pore size of the host is small. However, as the pores are filled up and effective pore size decreases, lithium metal deposition will shift outside the host, likely due to the wall pressure of the framework.^[^ ^8^ ^]^ Dendrite‐free lithium can be fully deposited inside a host with large pore size and embedded with nitrates. Previous work has established that desirable pore size is on the order of several micrometers.^[^ ^8^ ^]^ Quantitively, we have a targeted areal capacity of 5 mAh cm^−2^, a value commensurate with a practical cell design. For a carbon host with a porosity of 80% and assuming complete filling, the carbon host thickness will be 33 µm. Such an electrode will yield a specific capacity of 2000 mAh g^−1^ and 1500 mAh cm^−3^. Based on recent analysis, this anode performance will yield twice the gravimetric capacity of tin and twice the volumetric capacity of graphite.^[^ ^24^ ^]^

### 2.2 Effect of Embedded Nitrates on Li Plating in 3D Hosts

We first show that RbNO_3_ is a highly desirable choice to serve as a lithiophilic agent capable of regulating lithium deposition morphology in a 3D host. Here, we evaluated the ability of RbNO_3_ on enhancing lithiophilicity mainly based on the position of lithium deposition. Previously, LiNO_3_ has been widely used in ether and carbonate‐based electrolytes as nitrate are beneficial for SEI formation and dense lithium deposition morphology.^[^ ^18^, ^22^, ^25^, ^26^ ^]^ The reduction potential of the nitrate anion in the carbonate‐based electrolyte is ≈1.7 V versus Li/Li^+^, which means it will spontaneously react with lithium and repair any cracks in the SEI. Even at a low concentration of ≈800 ppm, nitrate is effective in improving lithium deposition morphology.^[^ ^18^ ^]^ Here, we chose RbNO_3_ due to its lower solubility in electrolyte than LiNO_3_. The solubility of RbNO_3_ in LEDV (1 M LiFSI in EC/DMC electrolyte with 5 wt% VC) was determined via inductively coupled plasma mass spectrometry (ICP‐MS). The concentration reaches ≈190 ppm in an hour and remains at this level thereafter (Figure S1, Supporting Information). This solubility is only about 25% of that of LiNO_3_. The use of RbNO_3_ allows us to reduce the amount of nitrate needed in the 3D host, key to maximizing electrode porosity and effective specific capacity.

We then fabricated a carbon host (SPR host) by casting a slurry of RbNO_3_, super P, and PVDF in a ratio of 1:1:1 wt%. In contrast, previous LiNO_3_ based 3D hosts (SPL host) contained 60 wt% of LiNO_3_. Also, a bare carbon host (SPC host) with super P and PVDF in a ratio of 1:1 wt% was fabricated to serve as a control.^[^ ^25^ ^]^ The areal mass of the SPR host is 1.34 mg cm^−2^. Based on the density and weight ratios, the electrode porosity of the SPR host is 66%, which can store 0.638 mg cm^−2^ Li, or 2.47 mAh cm^−2^. The theoretical volumetric capacity is 1357 mAh cm^−3^.

The cyclic voltammogram results of SPC, SPL, SPR hosts are shown in Figure S2, Supporting Information. The SPC host has only one peak (orange region) starting from ≈0.8 V, corresponding to EC reduction. In contrast, the SPL and SPR hosts have an additional peak (blue region) starting from ≈1.7 V, attributed to nitrate reduction.^[^ ^18^ ^]^ X‐ray photoelectron spectroscopy (XPS) analyses (Figure S3, Supporting Information) show the presence of Li_3_N, and Li*_x_* NO*_y_*_,_ and more LiF on the SPR host compared to the SPC host. The SEI components are consistent with those previously found in the presence of the LiNO_3_ additive (SPL host).^[^ ^22^ ^]^

Before we study the lithium deposition behavior in RbNO_3_‐loaded 3D hosts, we first examine the effect of RbNO_3_ on lithium deposition on Cu in LEDV at a current density of 0.5 mA cm^−2^ (Figure S4, Supporting Information). During the early stage of Li deposition, dendritic Li can be observed after 20 to 30 min on a RbNO_3_‐free host. In contrast, a dendrite‐free morphology is obtained in the presence of saturated RbNO_3_. This improvement in deposition shows that even at a concentration of 190 ppm, RbNO_3_ is sufficient to regulate Li deposition and achieve a similar morphology as those previously observed with LiNO_3_.

**Figure 2:**
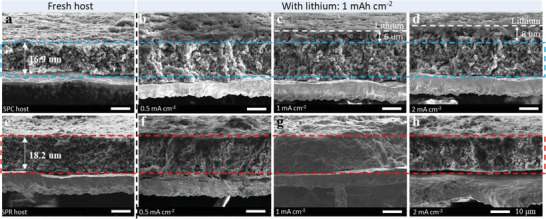
RbNO_3_ works as an additive in 3D lithium hosts. Cross‐sectional SEM images of pristine a) SPC host and e) SPR host. Cross‐sectional SEM images of 1 mAh cm^−2^ lithium deposition in b–d) SPC host and f–h) SPR host at a current density of 0.5, 1.0, and 2.0 mA cm^−2^, respectively.

We then evaluated the effect of RbNO_3_ on the position of lithium deposition and lithium morphology after 1 mAh cm^−2^ of lithium deposition at different current densities in 3D hosts (**Figure** **2** and Figure S5, Supporting Information). We found that the presence of RbNO_3_ had a significant effect on the lithium deposition behavior, and such effect was significantly enhanced at high current densities. At 0.5 mA cm^−2^, lithium is mainly deposited with a dendrite‐like morphology on the surface of the SPC host (Figure 2b and Figure S5b, Supporting Information). In contrast, lithium is deposited inside the SPR host (Figure 2f). Such a difference becomes more pronounced at 1 and 2 mA cm^−2^. As the current density increases, more lithium prefers to grow on the surface instead of in the pores of the SPC host (Figure 2b–d). In contrast, dendrite‐free lithium still grows in the pores of the SPR host, and there is no noticeable thickness change of the host at a current density up to 2 mA cm^−2^ (cross‐sectional view in Figure 2f–h and top view in Figure S5f–h, Supporting Information). We thus conclude that the embedded RbNO_3_ can optimize the lithiophilicity of the host, lithium morphology and guide lithium plating inside the 3D host.

### 2.3 Effect of Electrode Pore Structure on Li plating in 3D Hosts

**Figure 3:**
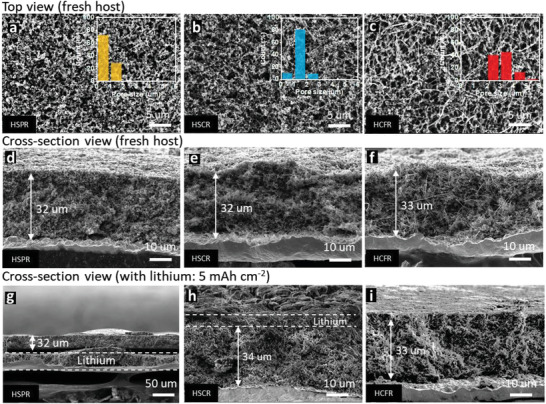
The effect of pore structure of the 3D host on Li plating. SEM images of a,d,g) HSPR host, b,e,h) HSCR host, and c,f,i) HCFR host, respectively. Embedded images are the corresponding pore size distribution of each host. a–c) and d–f) are the top view and the cross‐section view of fresh hosts, respectively. g–i) are the cross‐section view of hosts after 5 mAh cm^−2^ lithium plating at a current density of 1 mA cm^−2^ in LEDV. Note the difference in scalebars between (g) and (h–i).

We next examine the effect of electrode architecture on lithium plating behavior. Our approach is to use a mixture of super P and VGCF. By varying the ratio, we can readily tune the pore size distribution of the host. The hosts with a super P:VGCF ratio of 1:0, 1:1, and 0:1 wt% are referred to as HSPR, HSCR, and HCFR, respectively (**Figure** **3**a–c and Figures S6 and S7, Supporting Information; see Experimental Section). The pore size of each carbon host is defined as the shortest distance between two edges of a hole shown in SEM images, and the pore size distribution can be calculated based on 100 different spots via Image J analysis.^[^ ^27^ ^]^ Based on this method, the pore size distributions of the HSPR, HSCR, and HCFR host were found centered at ≈0.5 µm, ≈1.5 µm, and ≈3 µm, respectively (inset in Figure 3a‐c). All hosts have a thickness of ≈33 µm (Figure 3d–f) and contain ≈8 wt% RbNO_3_. The porosities are also similar, with values of 76%, 79%, and 80% for HSPR, HSCR, and HCFR hosts, respectively. Further, their electronic conductivities are all ≈0.05 S m^−1^. In order to achieve these high porosity values, LiPF_6_ was added as a pore former to the carbon slurry along with RbNO_3_. The LiPF_6_ was then removed by dissolution in DMC. As shown in Figure S6, Supporting Information, the removal of LiPF_6_ does not change electrode thickness. Based on their porosities, all hosts have a similar theoretical capacity at ≈5.4 mAh cm^−2^ (Equation S1, Supporting Information). The specific capacity of the HCFR host is 2018 mAh g^−1^ and 1643 mAh cm^−3^, which exceed our designed targets. X‐ray diffraction patterns of the baseline VGCF host and that with RbNO_3_ (HCFR host) show the presence of LiF in both hosts due to the thermo‐decomposition of LiPF_6_ during the thermal drying step. In addition, there are signals of RbNO_3_ and RbPF_6_ in the HCFR host (Figure S7, Supporting Information).^[^ ^28^ ^]^ The latter can be explained by the cation exchange between RbNO_3_ and LiPF_6_.

We then studied the electrode architecture influence on lithium plating in the 3D host. Here, 5 mAh cm^−2^ of lithium was deposited at a current density of 1 mA cm^−2^. In the HSPR host (pore size: ≈0.5 µm), the first 1 mAh cm^−2^ of lithium is deposited inside the host (Figure S8a, Supporting Information); however, additional lithium is deposited between the copper foil and the carbon host (Figure 3g), indicating that growth inside the small pores is no longer possible despite the lithiophilicity. It is worth mentioning that the lithium deposited outside the host still exhibits dendrite‐free morphology (Figure S8b, Supporting Information). In the HSCR host (pore size: ≈1.5 µm), lithium is initially plated inside the 3D host, up to 3 mAh cm^−2^ (Figure S8c,d, Supporting Information). When the capacity reaches 5 mAh cm^−2^, around 1 mAh cm^−2^ of lithium (≈5 µm in thickness) can be found on the surface of the host (Figure 3h). The larger pore‐size host allows more lithium deposition inside the host. In the HCFR host (pore size: ≈3 µm), all the lithium (5 mAh cm^−2^) is plated inside the 3D host (Figure 3i). There is no lithium deposited under (Figure S8g–i, Supporting Information) or on top of the host (Figure S9, Supporting Information). The results indeed show that the pore structure of the 3D host has a significant effect on guiding the lithium deposition in the pores of the 3D host, even when these hosts have almost the same theoretical capacity, porosity, and chemical composition.

### 2.4 Electrochemical Performance of Optimized 3D Hosts

The above morphological studies revealed that the embedded nitrate additives and the pore size distribution of the 3D host both play essential roles in guiding the lithium deposition in the pores of the 3D host. We next quantitatively evaluated the optimized host in multiple electrolytes. In Li/Cu half‐cell tests at a current density of 1 mA cm^−2^ for 1 mAh cm^−2^ with a carbonate electrolyte (LEDV, Figure S10, Supporting Information), Cu foil exhibited a poor CE at ≈92%, while the VGCF host delivered a slightly better CE at ≈96% during the first 30 cycles but failed with rapid deterioration of CE soon after. When the Cu foil electrode was evaluated with the same carbonate electrolyte with saturated RbNO_3_ (≈190 ppm), it exhibited an increased CE at ≈98% for the first 75 cycles followed by continuous decay, indicating potential exhaustion of nitrate ions. In contrast, the HCFR host (with 8 wt% embedded RbNO_3_) exhibited a stable and high CE at ≈98% over 125 cycles. RbNO_3_ can still be detected in the cycled electrode so that the exhaustion of nitrate anion during cycling was not encountered (Figure S11, Supporting Information). Hence, both the 3D structure and the embedded nitrates are essential components of the HCFR host, which together can improve the CE and cycling stability of lithium anodes. A full cell with LEDV was also fabricated with either Cu or HCFR as the anode. The cell with HCFR demonstrated a capacity retention of 92% as compared to 27% after 50 cycles (Figure S12, Supporting Information). Hence, regulating the position of lithium deposition and lithium morphology in 3D anode are conducive to mitigating the electrochemical degradation due to the poor plating‐stripping behavior of the lithium metal anode.

**Figure 4:**
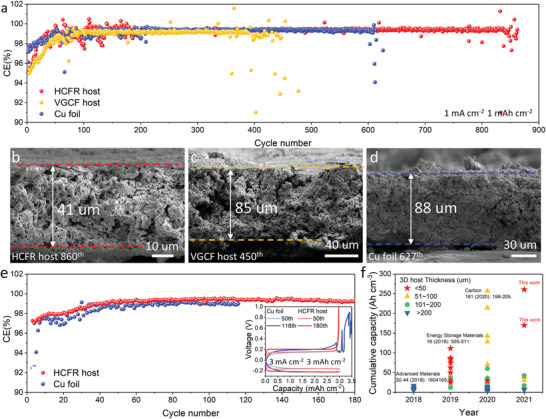
Electrochemical performance of HCFR host in LDME electrolyte with saturated RbNO_3_. a) Coulombic efficiency of HCFR host, VGCF host, and Cu foil, respectively, at a current density of 1 mA cm^−2^ for 1 mAh cm^−2^; Cross‐sectional SEM images of cycled b) HCFR host, c) VGCF host, and d) Cu foil; e) Coulombic efficiency and the Li plating/stripping voltage profiles (inset) of HCFR host, Cu foil, respectively, at a current density of 3 mA cm^−2^ for 3 mAh cm^−2^. f) Comparison of 3D lithium anode electrochemical performance. Cumulative capacity is capacity per volume per cycle multiplied by cycle number.

We further evaluated the electrochemical performance of the HCFR host with an ether‐based electrolyte (2 M LiFSI/DME‐BTFE (1:4, w/w), referred to as LDME, with saturated RbNO_3_). LDME was previously reported as a novel ether‐based localized high concentration electrolyte for high‐performance lithium metal anodes.^[^ ^25^ ^]^ With this electrolyte, lithium can also be plated inside the HCFR host at 1 mA cm^−2^ for 5 mAh cm^−2^ (Figure S13, Supporting Information). In half‐cell tests (**Figure** **4**a), the HCFR host exhibited a CE of 99.36% over 860 cycles at 1 mA cm^−2^ for 1 mAh cm^−2^, which was more stable than the VGCF host (≈450 cycles), and Cu foil (≈600 cycles), a trend similar to that observed in carbonate electrolytes. Interestingly, all three anodes exhibited a similar CE over 99% but with different cycling lives, which is likely due to the significant difference in the rate of increase of anodic thickness during cycling. SEM images (Figure 4b–d) show that the thickness of the cycled VGCF host was 85 µm (versus 32 µm before cycling), where dead lithium mainly accumulated between the host and the separator. The thickness of deposited Li on cycled Cu foil was around 90 µm. In contrast, the thickness of the cycled HCFR host was only 41.3 µm (an increase of 8.3 µm from 33 µm), demonstrating that both electrode architecture and the embedded nitrate played critical roles in suppressing the growth of dead lithium and improving anode cycling stability. We also performed a further evaluation of the Cu foil and the HCFR host at a high current density of 3 mA cm^−2^ for 3 mAh cm^−2^, where Cu foil failed in 118 cycles, but HCFR host still delivered a high CE at 99.05% over 180 cycles (Figure 4e).

To properly compare the performance of different 3D hosts, one needs to consider its specific and volumetric capacity, the utilization of the porosity during cycling, and the cycling stability. Here we advocate using cumulative capacity per unit volume as a comprehensive metric to facilitate this comparison. A summary of the 3D anode cumulative capacity is presented in Figure 4f, with details listed in Table S1, Supporting Information. One report in 2018 added silver wires into graphene hosts that can facilitate Li nucleation and suppress dendrite formation. However, the graphene host itself was thick, >200 µm, and the porosity utilization of the host was low, resulting in a low specific cumulative capacity.^[^ ^12^ ^]^ In 2019, there was a rapid improvement of specific cumulative capacity due to the development of thin 3D hosts (<50 µm). For example, Liu et al. reported an 18‐µm‐thick 3D host with embedded LiNO_3_ that cycled at 1 mA cm^−2^ and 1 mAh cm^−2^ over 200 cycles, where the cumulative capacity of the 3D host exceeded 100 Ah cm^−3^ for the first time.^[^ ^22^ ^]^ In 2020, high‐performance electrolytes (CE > 99%) and high‐porosity electrodes enabled the cumulative capacity to exceed 250 Ah cm^–3^.^[^ ^29^ ^]^ However, the fabrication processes of those hosts were usually complicated and expensive, such as de‐alloying, freeze drying, and electrospinning.^[^ ^9^, ^29^, ^30^ ^]^ In this work, we have presented a simple but effective slurry‐casting method to fabricate thin 3D lithium hosts, which has delivered an average coulombic efficiency of 99.36% at 1 mA cm^−2^ for 1 mAh cm^−2^ over 860 cycles, where the cumulative capacity has reached 260 Ah cm^−3^, making it a great candidate to fabricate high energy density LMBs.

**Figure 5:**
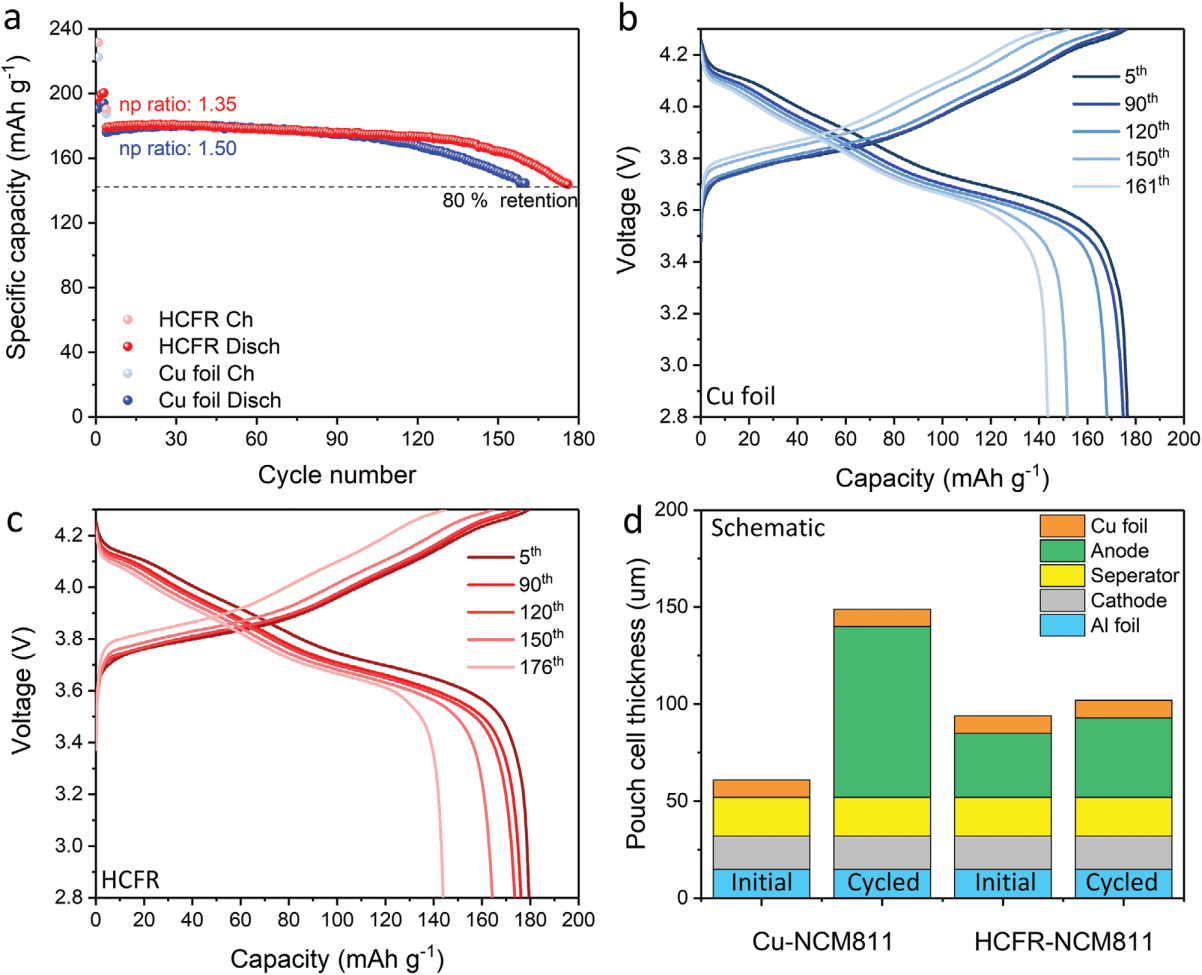
Full‐cell electrochemical performance of HCFR host paired with NMC811 in LDME electrolyte with saturated RbNO_3_. a) Full cell tests of Cu foil and HCFR host with NMC811 as cathode cycled at C/2, e/c ratio is 3 g Ah^−1^. b,c) are the voltage profiles of Cu foil‐NMC811 and HCFR‐NMC811 cells, respectively. The voltage range is 2.8−4.3 V. d) Comparison of pouch cell thickness variation before and after cycling with Cu or HCFR as the anode assuming a capacity of 1 mAh cm^−2^.

Cell swelling is considered a key challenge in practical LMBs.^[^ ^30^, ^31^ ^]^ Because of significant volume expansion, the electrolyte cannot thoroughly wet both the incompact lithium and the active cathodic materials to continue electrochemical reactions, especially in lean electrolyte conditions.^[^ ^30^ ^]^ Here, we evaluated full cells made of Cu foil and the HCFR host with 2 mAh cm^−2^ pre‐deposited lithium as the anode, paired with NMC811 as the cathode (mass loading: ≈7 mg cm^−2^), under a lean electrolyte condition of 3 g Ah^−1^. After three conditioning cycles at C/10, the cells were cycled at C/2 until the capacity retention reached 80% (**Figure** **5**a‐c). The Cu‐NMC811 cell exhibited a capacity of 1.33 mAh cm^−2^ (N/P ratio: 1.50). The cell lasted 161 cycles, where the average CE for the Li anode was calculated to be 98.9%. In comparison, the HCFR‐NMC811 cell exhibited a capacity of 1.48 mAh cm^−2^ (N/P ratio: 1.35) cycled for 176 cycles, with an average CE of 99.1% for the anode. Using the anti‐swelling anode helps to mitigate the electrochemical degradation due to poor wetting, directly leading to the improvement on the anode CE.

Though LMBs exhibit high potential to achieve higher energy densities than those of Li‐ion batteries, the cycling stability and safety concerns of this battery technology are far from being solved.^[^ ^31^, ^32^ ^]^ More study is needed on the details of LMBs degradation, especially the Li metal plating‐stripping behavior. Here, it is worthwhile to schematically summarize the major finding of LMBs with the HCFR host (Figure 5d). A thickness‐evolution model of lithium‐free pouch cell was built based on our experimental observation from the half‐cell test, 1 mA cm^−2^ for 1 mAh cm^−2^ (Figure 4d). In order to match the same areal capacity, an NMC811 cathode, 1 mAh cm^−2^, is paired with Cu foil (Cu‐NMC811) and the HCFR host (HCFR‐NMC811). After cycling, the swelling ratio of HCFR‐NMC811 is only 8.5% (860 cycles), while the swelling ratio reaches 144% in Cu‐NMC811 (627 cycles), assuming the increasing rate of anodic thickness is similar between half‐cell test and full‐cell test. This will lead to a significant difference in electrochemical performance due to the electrolyte wetting issue. In this regard, this work not only generates optimum lithium morphology via adding RbNO_3_, but also by mitigating the swelling issue for better cycling stability via anode architecture engineering.

## 3 Conclusion

We have described a high‐porosity VGCF framework with embedded RbNO_3_ as a practical high‐capacity 3D lithium host fabricated by a slurry‐casting method. The HCFR host with only 8 wt% embedded RbNO_3_ achieved similar control over lithium morphology and electrochemical performance as the SPL host previously fabricated with 60 wt% embedded LiNO_3_. Additionally, the pore size of the 3D host is important. Lithium is deposited uniformly inside the 3D host when the pore size of the host is at least 3 µm. The HCFR host with high porosity of 80% possesses a volumetric capacity of 1643 mAh cm^−3^. With an N/P ratio of 1.35 and an E/C ratio of 3 g Ah^−1^, the HCFR||NMC811 cell achieves 80% capacity retention when cycled at a C/2 rate for 176 cycles. This work delineates the roles of pore size and host lithiophilicity for developing high‐capacity 3D lithium hosts for practical applications in batteries with lean electrolyte amounts.

## Conflict of Interest

The authors declare no conflict of interest.

## Supporting information

Supporting Information

## Data Availability

The data that support the findings of this study are available from the corresponding author upon reasonable request.
