# Supplementary material for: A Fiber‐Based 3D Lithium Host for Lean Electrolyte Lithium Metal Batteries
Source: Adv Sci (Weinh). 2022 Feb 1;9(10):2104829. doi: 10.1002/advs.202104829 (PMC8981896; doi:10.1002/advs.202104829)
Supplement: Supplementary file 1 — Supporting Information [file ADVS-9-2104829-s001.pdf]

ADVANCED SCIENCE
Open Access

Supporting Information

for *Adv. Sci.*, DOI 10.1002/advs.202104829

A Fiber-Based 3D Lithium Host for Lean Electrolyte Lithium Metal Batteries

*Sicen Yu, Zhaohui Wu, John Holoubek, Haodong Liu, Emma Hopkins, Yuxuan Xiao, Xing Xing, Myeong Hwan Lee and Ping Liu**

Supporting Information

for *Adv. Sci.*, DOI: 10.1002/advs.202104829

A Fiber-based 3D Lithium Host For Lean Electrolyte Lithium Metal Batteries

*Sicen Yu, Zhaohui Wu, John Holoubek, Haodong Liu, Emma Hopkins, Yuxuan Xiao, Xing Xing, Myeong Hwan Lee, Ping Liu**

Experimental Section

1. 3D composite electrode preparation

Rubidium nitrate ($RbNO_3$), lithium nitrate ($LiNO_3$) and vapor-growth carbon fiber (VGCF) were purchased from Sigma-Aldrich. Super P carbon (Super P) was received from TIMCAL. Poly(vinylidene fluoride)-1800 (PVDF1800) and Poly(vinylidene fluoride)-900 (PVDF900) were received from Kynar. N-methylpyrrolidone (NMP) was purchased from ACROS Organics. Lithium hexafluorophosphate ($LiPF_6$) was purchased from BASF. The seven 3D composite electrodes of $LiNO_3$:SuperP:PVDF = 3:1:1 wt% (SPL host), $RbNO_3$:Super P:PVDF1800 = 1:1:1 wt% (SPR host), SuperP:PVDF1800 = 1:1 wt% (SPC host), $RbNO_3$:SuperP:$LiPF_6$:PVDF900 = 3:15:50:20 wt% (HSPR), $RbNO_3$:VGCF:SuperP:$LiPF_6$:PVDF900 = 3:7.5:7.5:50:20 wt% (HSCR), $RbNO_3$:VGCF:$LiPF_6$:PVDF900 = 3:15:50:20 wt% (HCFR), and

VGCF:$LiPF_6$:PVDF900 = 15:50:20 wt% (VGCF host) were prepared by slurry coating method. The slurries were prepared by mixing predetermined amounts of materials with NMP, and then cast them each onto a Cu foil using a doctor blade and dried in an oven overnight at 80 °C. For HSPR, HSCR, and HCFR, dried electrodes were rinsed by dimethyl *carbonate* (99%, Sigma) and dried in the glovebox antechamber under vacuum (MTI corporation). All processes were operated inside the glovebox filled with Ar.

2. Porosity calculation

The theoretical porosity of the 3D host can be calculated via porosity equation:

$$\text{Porosity} = \frac{V_{electrode} - \sum V_{component}}{V_{electrode}} = \frac{V_{electrode} - M_{electrode} \cdot \left(\frac{P_R}{\rho_R} + \frac{P_C}{\rho_C} + \frac{P_{PVDF}}{\rho_{PVDF}}\right)}{V_{electrode}} \quad (1)$$

where $V_{electrode}$ and $V_{component}$ are the volumes of electrode and components ($RbNO_3$, Super P, and PVDF), and P and ρ are the mass fraction of components and each density, respectively. In this experiment, the densities of $RbNO_3$, carbon (Super P & VGCF), and PVDF are 3.11, 2.0, and 1.76 g cm^{-3}, respectively.

3. Electrolyte preparation

Battery grade vinylene carbonate (VC), ethylene *carbonate* (EC), and dimethyl *carbonate* (DMC) were acquired from Shenzhen CAPCHEM Technology Co. Ltd. The 1,2-dimethoxyethane (DME) was purchased from Gotion. The bis(2,2,2-trifluoroethyl)ethe (BTFE), and Lithium bis(fluorosulfonyl)imide (LiFSI) were purchased from Sigma Aldrich. The four electrolytes of 1 M LiFSI/EC-DMC (1:1, w/w) with 5 wt% VC, 1 M LiFSI/EC-DMC (1:1, w/w) with 5 wt% VC and saturated $RbNO_3$, 2 M LiFSI/DME-BTFE (1:4, w/w) and 2 M LiFSI/DME-

BTFE (1:4, w/w) with saturated $RbNO_3$ were prepared by dissolving predetermined amounts of LiFSI salt into mixed solvents and stirred to achieve stable solutions. Here, 1 M is defined as 1 M salt dissolved into 1 kg of solvent.

4. Electrochemical test

2032-type coin cells were used for all the electrochemical studies in this work. The 250 μm thick lithium was punched to 12 mm discs as the counter electrode. The Celgard 25 μm trilayer PP-PE-PP membrane was used as a separator.

The 250 μm thick lithium was punched to 12 mm discs as the Li electrode for half-cell tests. The 9 mm thick Cu foil and as-prepared 3D hosts were punched to 14 mm discs as the current collector. A fixed amount of electrolyte (50 μL) was added into each coin cell to guarantee the complete wetting of the separator and electrodes. Galvanostatic cycling was conducted on an LBT-5V5A battery tester (Arbin instruments) and Land battery tester. The cycled electrode was recovered by disassembling the coin cell. All the samples were washed with DMC three times and dried in the glovebox antechamber under vacuum.

For $LiNi_{0.8}Mn_{0.1}Co_{0.1}O_2$ (NMC811) full-cell tests, the Cu foil and as-prepared 3D hosts were punched to 14 mm discs as the current collector, the self-made NMC811 electrode (NMC811: super P: polyvinylidene fluoride (PVDF-1800) = 96:2:2, wt%) was punched to 12 mm discs as the cathode. The cathode loading is around 7 mg cm^{-2}. The electrolyte/capacity ratio is 3 g Ah^{-1}. Galvanostatic charge/discharge was conducted between 2.8 and 4.3 V. Galvanostatic cycling was conducted on an LBT-5V5A battery tester (Arbin instruments). The cycled electrode was recovered by disassembling the coin cell. All the samples were washed with DMC three times and dried in the glovebox antechamber under vacuum.

5. Scanning electron microscopy

The morphology and thickness of the deposited Li metal film and 3D composite electrode were characterized using scanning electron microscopy (FEI Apreo). The sample was adhered to a double-sided carbon tape and placed on a specimen holder. The prepared sample was sealed in a plastic laminate bag inside the glovebox for transferring to the SEM. The approximate time of sample exposed to air (from a sealed environment to the SEM stage) was less than 3 s.

6. X-ray diffraction

The crystal structure of materials of interest were identified by X-ray diffraction (XRD), acquired using a Bruker D2 phaser diffractometer with a Bragg- Brentano θ-2θ geometry and a Cu Kα source (λ = 1.54 Å). Air-sensitive samples were sealed inside the glovebox by Kapton tape, which was scanned from 10° to 80° at a scan rate of 0.02° s^{-1}.

7. X-ray photoelectron spectroscopy

The surface analysis of materials of interest was identified by X-ray photoelectron spectroscopy (XPS, PHI Quantera SXM). XPS was carried out using an Al anode source at 15 kV, and all the peaks were calibrated based on the reference C–C bond at 284.8 eV. All XPS measurements were collected using a charge neutralizer during acquisition. Survey scans were collected with a step size of 1.0 eV followed by high-resolution scans with a step size of 0.125 eV for the C 1s, N 1s, and F 1s regions. The sample was adhered to a double-sided carbon tape and placed on a sample stage. The prepared sample was sealed in a plastic laminate bag inside the glovebox for transferring to the XPS. The approximate time of

sample exposed to air (from a sealed environment to the intro chamber of XPS) was less than 3 s.

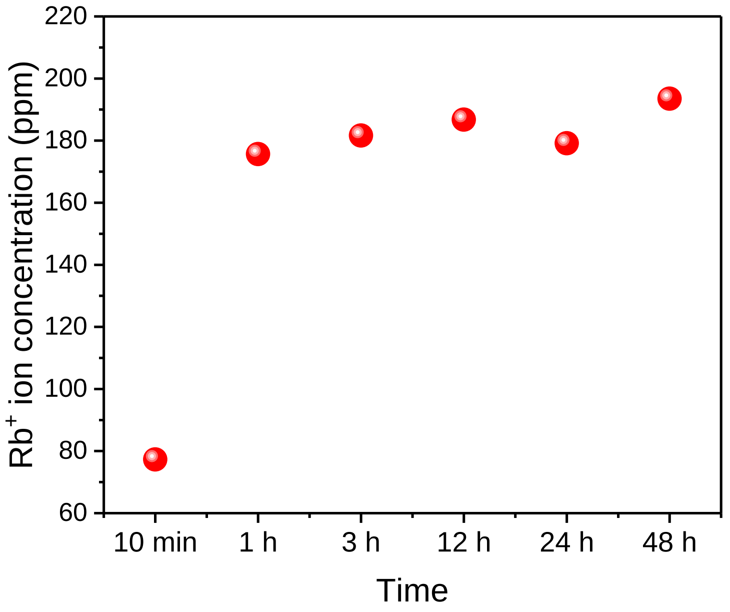

Figure S1. The concentration of rubidium ion in a carbonate electrolyte (1 M LiFSI/EC-DMC (1:1, w/w) with 5 wt% VC), determined by ICP-MS;

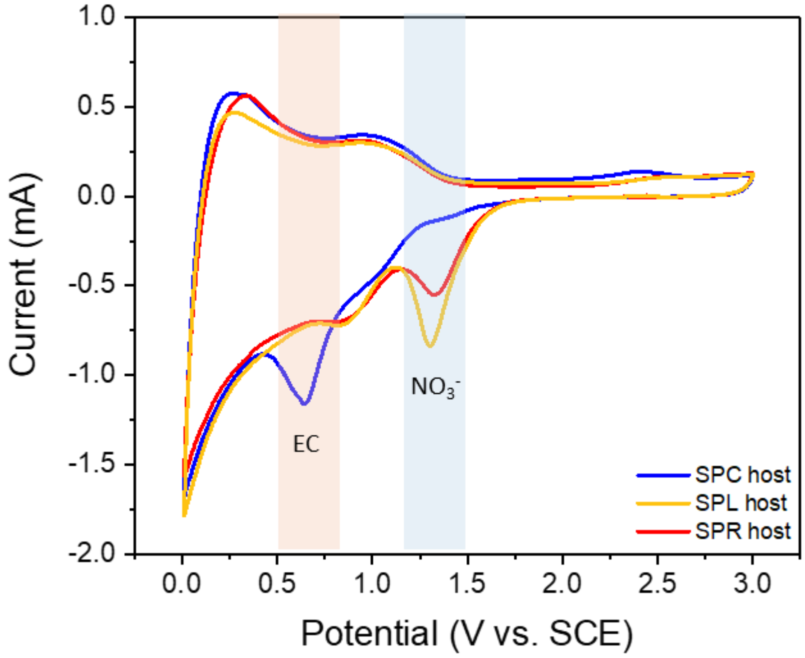

Figure S2. CV results of the SPC host, SPL host, and SPR host in a carbonate electrolyte (1 M LiFSI/EC-DMC (1:1, w/w) with 5 wt% VC) at a scan rate of 1 mV s^{-1}. Blue region stands for nitrate ion reduction. Orange region stands for EC reduction.

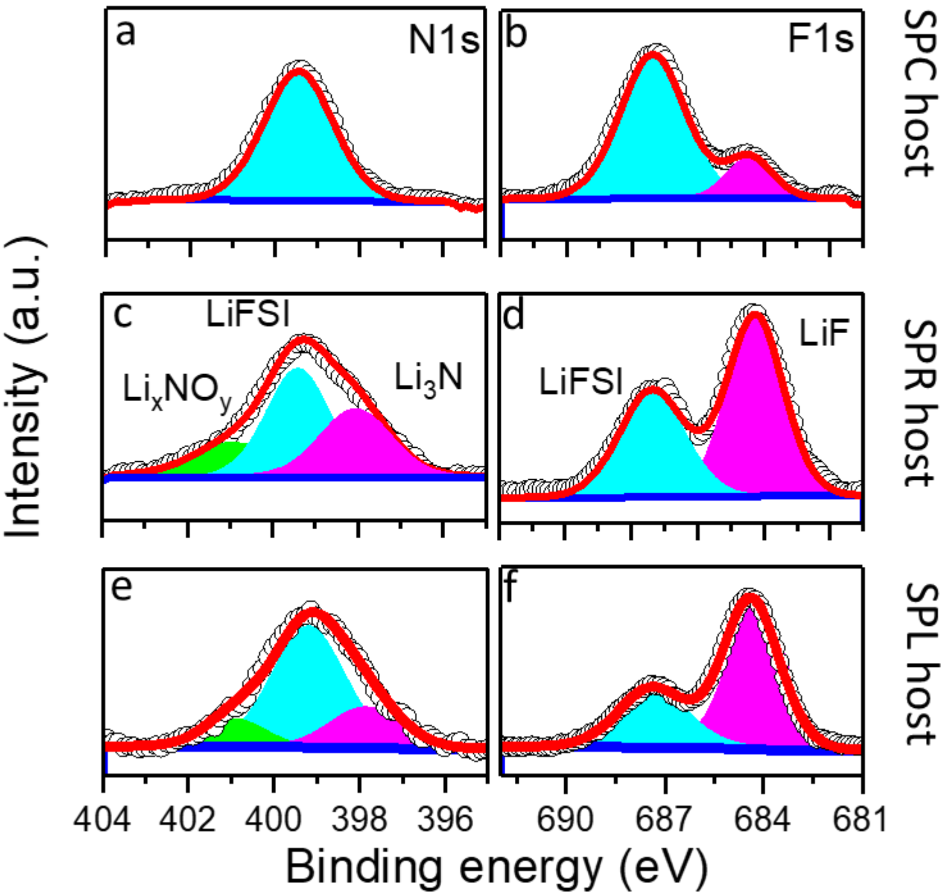

Figure S3. XPS results of the SPC host, SPL host, and SPR host after the first cycle, respectively. 1 M LiFSI/EC-DMC (1:1, w/w) with 5 wt% VC.

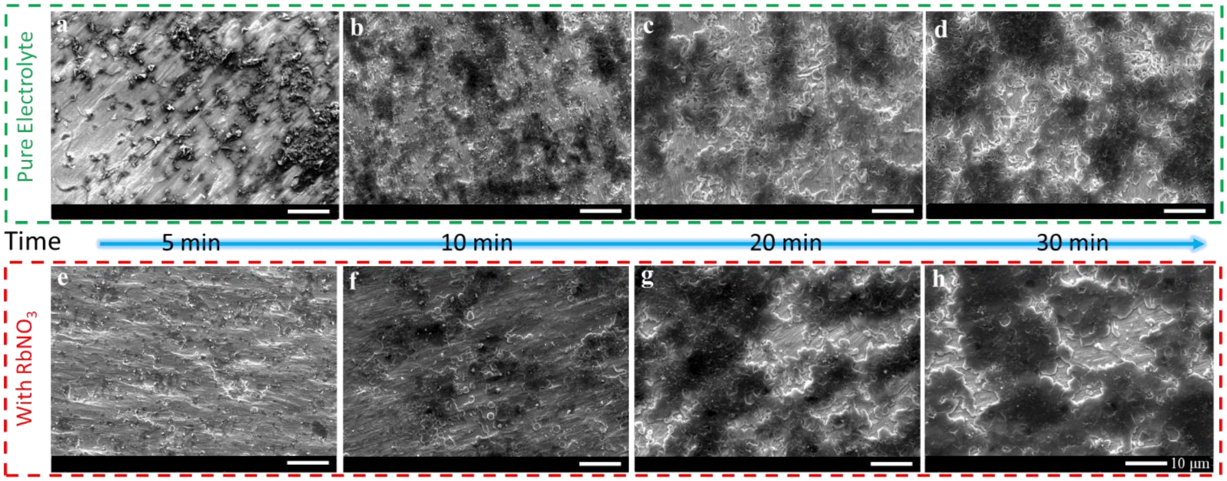

Figure S4. SEM images of the deposited Li metal film (a) 5 min, (b) 10 min, (c) 20 min, and (d) 30 min on Cu foil at a current density of 0.5 mA cm^{-2} in 1M LiFSI in EC/DMC electrolyte with 5wt% VC. SEM images of the deposited Li metal film (e) 5 min, (f) 10 min, (g) 20 min, and (h) 30 min on Cu foil at a current density of 0.5 mA cm^{-2} in LEDV.

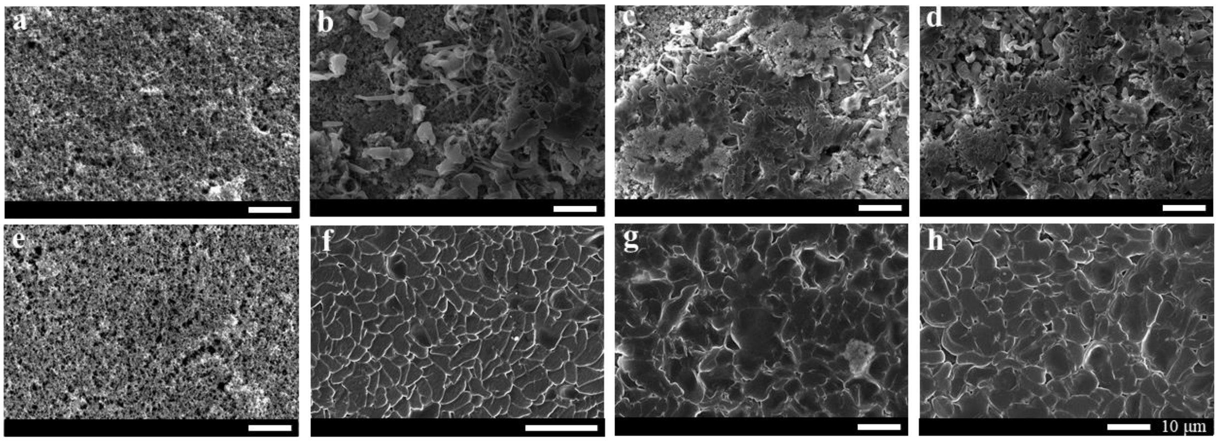

Figure S5. $RbNO_3$ works as additives in the 3D lithium host. SEM images (top view) of pristine (a) SPC host and (e) SPR host. SEM images of 1 mAh cm^{-2} lithium deposition in (b-d) SPC host and (f-h) SPR host at a current density of 0.5 mA cm^{-2}, 1.0 mA cm^{-2} and 2.0 mA cm^{-2}, respectively.

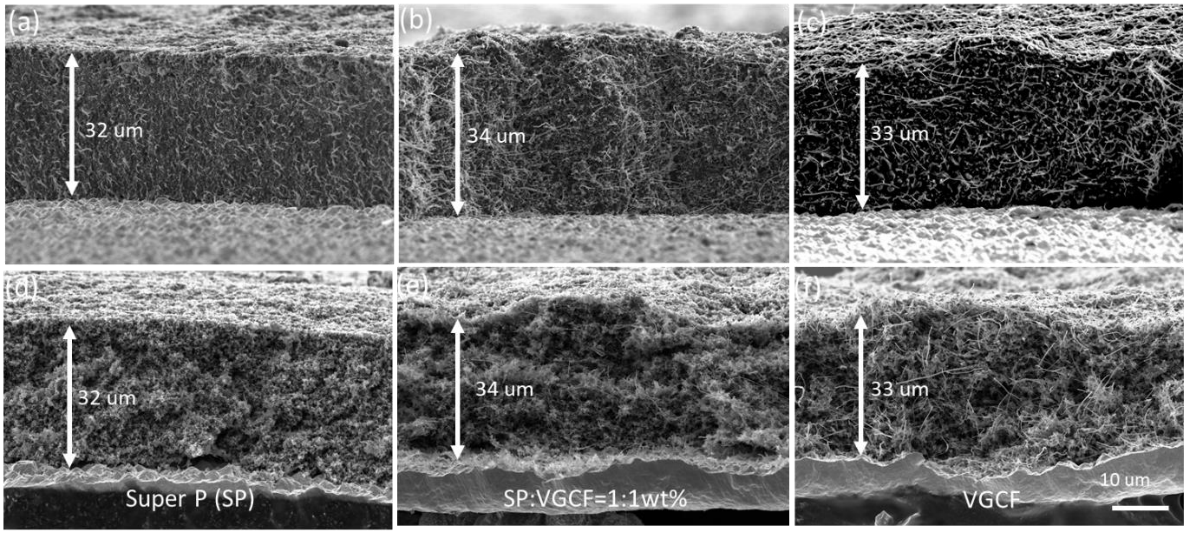

Figure S6. SEM images of high porosity 3D host. (a)(d) HSPR host, (b)(e) HSCR host, and (c)(f) HCFR host. (a-c) are unwashed samples. (d-f) are washed samples.

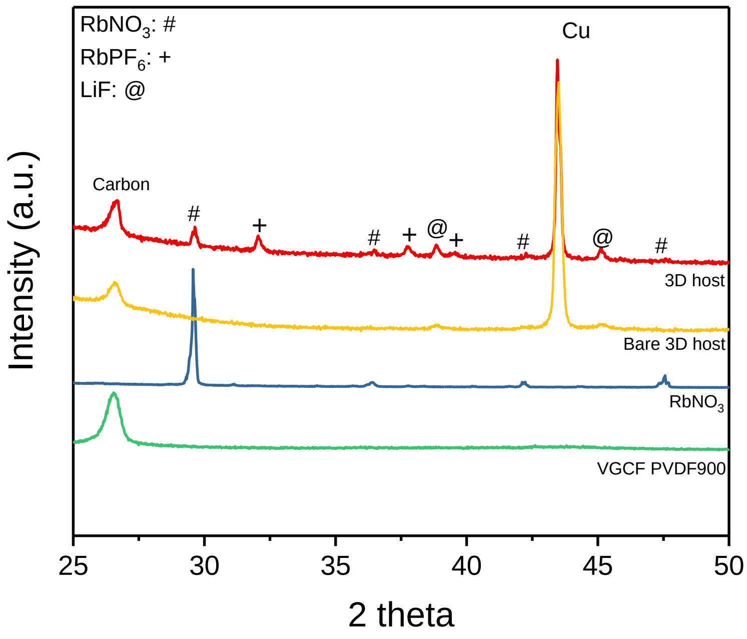

Figure S7. The XRD evolution of HCFR host fabrication.

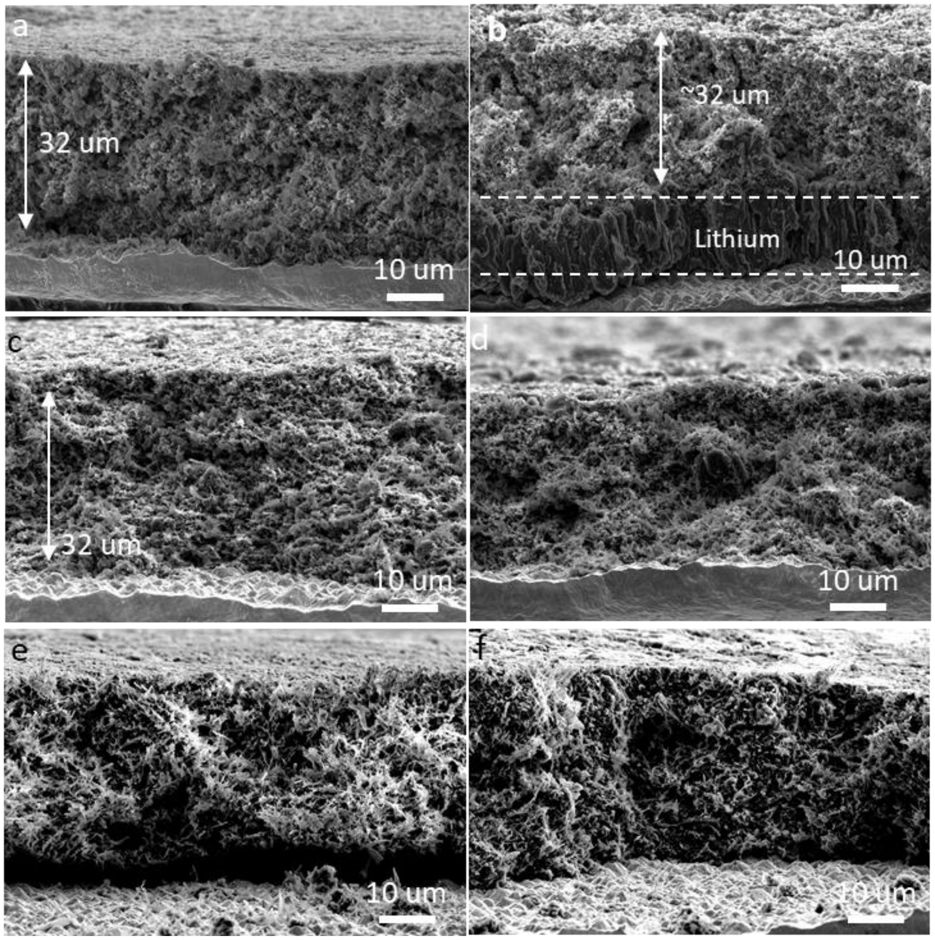

Figure S8. The geometry influence on Li plating. SEM images of lithium plating in (a-c) HSPR host, (d-f) HSCF host, and (g-i) HCFR host at a current density of 1 mA cm^{-2} for 1 mAh cm^{-2} and 3 mAh cm^{-2}, respectively, in 1 M LiFSI EC/DMC electrolyte with 5 wt% VC.

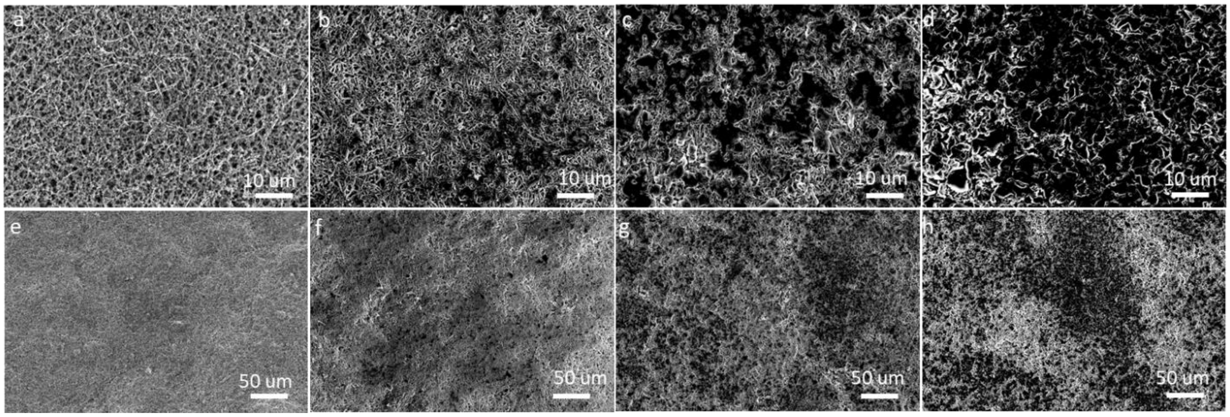

Figure S9. SEM images of lithium plating in HCFR host. (ae) fresh samples, after lithium plating at a current density of 1 mA cm^{-2} for (bf) 1 mAh cm^{-2}, 3 mAh cm^{-2}, 5 mAh cm^{-2}, respectively, in 1 M LiFSI EC/DMC electrolyte with 5 wt% VC.

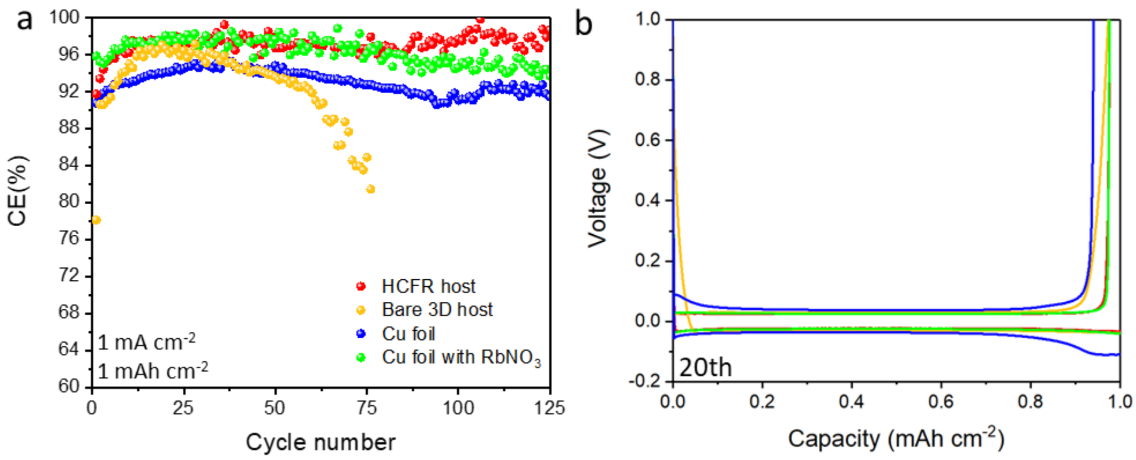

Figure S10. Electrochemical performance of HCRF host in 1 M LiFSI EC/DMC electrolyte with 5 wt% VC. (a) Half-cell tests of $RbNO_3$ host, bare host, Cu foil, and Cu foil with $RbNO_3$, respectively, at a current density of 1 mA cm^{-2} for 1 mAh cm^{-2}. (b)The Li plating/stripping voltage profiles on HCFR host, bare 3D host, Cu foil, and Cu foil with $RbNO_3$, respectively.

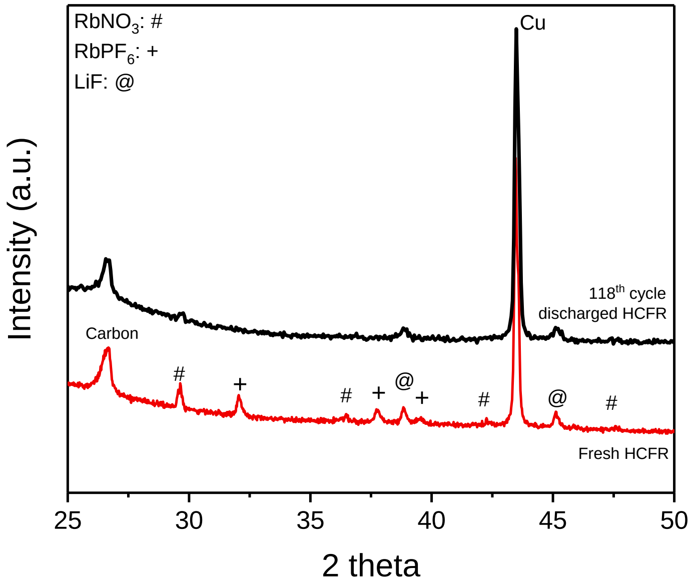

Figure S11. XRD pattern of cycled HCFR host.

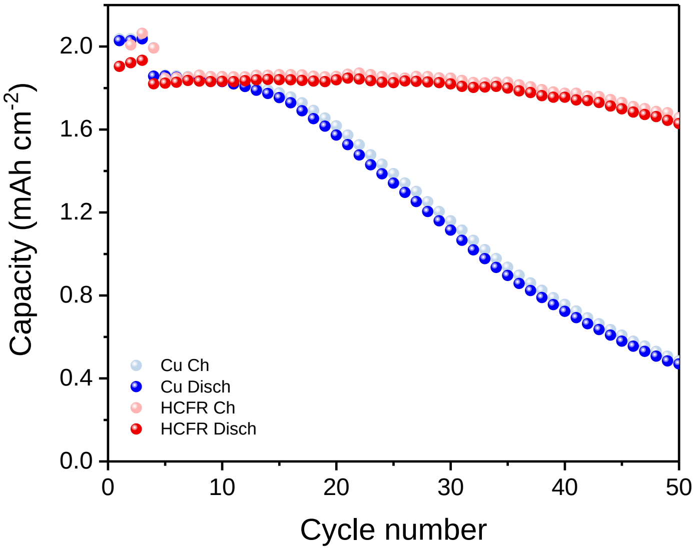

Figure S12. Full-cell electrochemical performance of Cu foil and HCFR host paired with NMC811 in LEDV electrolyte with saturated $RbNO_3$. We have also evaluated full cells made of 50 uL 1M carbonate electrolyte with Cu foil or the HCFR host with 1 mAh cm^{-2} pre-deposited lithium as the anode, paired with ~10 mg cm^{-2} NMC811 as the cathode (N/P ratio: 0.5). After

three conditioning cycles at C/10, the cells were cycled at C/2. After 50 cycles, the capacity retention of the Cu-NMC811 cell is 27%, where the average CE of the anode is only 96.33%. In contrast, the capacity of the HCFR-NMC811 cell only reduced from 1.8 mAh cm^{-2} to 1.65 mAh cm^{-2}, where the average CE of the anode is 98.7%. Hence, using the HCFR host is conducive to mitigating the electrochemical degradation via optimizing lithium plating-stripping behavior on anode for this diluted solution condition.

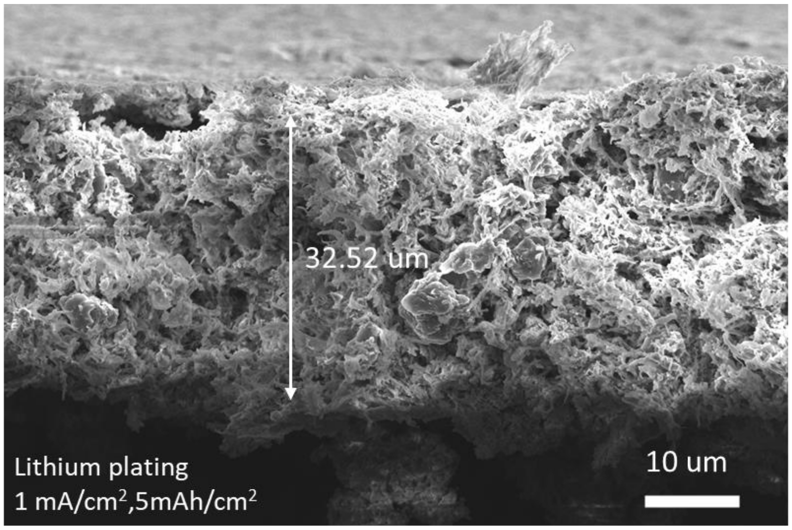

Figure S13. SEM image of the cross-section view of HCFR host after 5 hours Li deposition at 1 mA cm^{-2}.

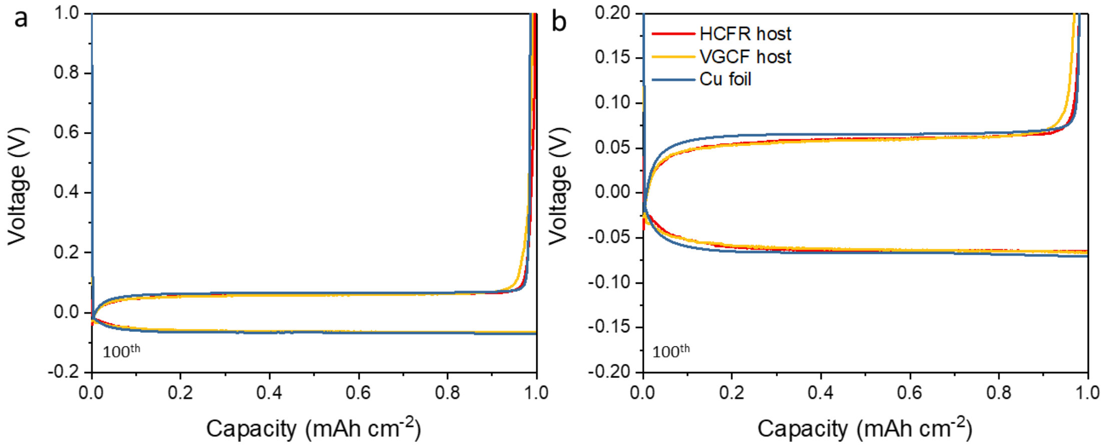

Figure S14. The 100th Li plating/stripping voltage profiles on HCFR host, bare 3D host, and Cu foil, respectively, at a current density of 1 mA cm^{-2} for 1 mAh cm^{-2} in LDME electrolyte with saturated $RbNO_3$, and Cu foil at a current density of 1 mA cm^{-2} for 1 mAh cm^{-2} in LDME

Table S1. Summary of 3D lithium anode coulombic efficiencies.

Tile of literature	Materials	Thickness (um)	Current (mA cm^{-2})	Capacity (mAh cm^{-2})	Cycle #	Cumulative Capacity (mAh cm^{-3})	CE
A Hierarchical Silver-Nanowire–Graphene Host Enabling Ultrahigh Rates and Superior Long-Term Cycling of Lithium-Metal Composite Anodes (2018)	Ag wire	350	1	6	50	8571.429	97.3
			1	12	50	17142.86	97.6
Ultrafine Titanium Nitride Sheath Decorated Carbon Nanofiber Network Enabling Stable Lithium Metal Anodes (2019)	CNF-TiN	40	1	1	300	75000	95.8
			2	1	250	62500	96.8
			3	1	200	50000	96.8
			4	1	120	30000	98.2
A 3D Lithiophilic Mo2N-Modified	Mo_2N-CNF	120	1	1	150	12500	99.6

Carbon Nanofiber Architecture for Dendrite-Free Lithium-Metal Anodes in a Full Cell (2019)			4	3	150	37500	99.2
A scalable 3D lithium metal anode (2019)	Super P	17.9	0.25	0.5	300	83798.88	98.4
			1	1	200	111731.8	97.9
			2	2	100	111731.8	97.1
Toward real-time monitoring of lithium metal growth and dendrite formation surveillance for safe lithium metal batteries (2020)	CNF	120	1	3	140	35000	97.5
Tortuosity Effects in Lithium-Metal Host Anodes (2020)	Graphene	125	1	1	300	24000	99.1
			5	5	150	60000	99.08
			3	3	150	36000	96
Inducing uniform lithium nucleation by integrated lithium-rich li-in anode with lithiophilic 3D framework (2020)	Li-In	17	0.5	0.5	100	29411.76	95
**VGCF 3D conducting host coating on glass fiber filters for lithium metal anodes (2018)**	VGCF	400	0.5	2	100	5000	89.3
			0.5	2.5	200	12500	91.1
			0.5	3	100	7500	91.7
Stable Lithium Metal Anode Enabled by 3D Soft Host (2020)	AgNWs	600	0.5	1	300	5000	99.1
			1	1	180	3000	99.1
			2	1	110	1833.333	98.8
Gradient□Distributed Nucleation Seeds on Conductive Host for a Dendrite□Free and High□Rate Lithium Metal Anode **(2019)**	CNF-ZnO	40	0.5	0.5	700	87500	98.1
			1	0.5	200	25000	96.8
			3	0.5	200	25000	96.1
			5	0.5	200	25000	96
Redox-Driven Lithium Perfusion to Fabricate Li@Ni−Foam Composites for High Lithium-Loading 3D Anodes **(2020)**	NiO-Ni foam	1000	1	1	200	2000	99
Polypyrrole Nanotube Sponge Host for Stable Lithium-Metal Batteries under Lean Electrolyte Conditions **(2021)**	Polypyrrole nanotube sponge	65	0.5	0.5	500	38461.54	98.7
			1	1	200	30769.23	98.1
			2	2	100	30769.23	97.8
Spatially uniform Li deposition realized by 3D continuous duct-like graphene host for high energy density Li metal anode **(2020)**	Graphene	70	2	2	500	142857.1	98.7
			2	4	225	128571.4	99
			2	20	90	257142.9	99
			5	10	150	214285.7	98.7
			10	5	100	71428.57	98.7
Restructured rimous copper foam as robust lithium host **(2020)**	Cu Foam	500	1	1	660	13200	99
			2	1	380	7600	98.7
			3	1	220	4400	98.8
			1	2	220	8800	98.3
			1	4	180	14400	99.7
A Fishing□Net□Like 3D Host for Robust and Ultrahigh□Rate Lithium	MgZnO/CNF□rGO	118	1	1	500	42372.88	95

			5	1	200	16949.15	95
Self-Standing N-Doped Carbonized Cellulose Fiber as a Dual Functional Host for Lithium Metal Anodes **(2021)**	Nitrogen-doped carbonized cellulose fiber	390	1	3	100	7692.308	96
			1	5	50	6410.256	95
Polypyrrole Nanotube Sponge Host for Stable Lithium-Metal Batteries under Lean Electrolyte Conditions **(2021)**	Polypyrrole nanotube	65	0.5	0.5	500	38461.54	98.7
			1	1	200	30769.23	98.1
			2	2	100	30769.23	97.8
**This work**	VGCF	33	1	1	860	260606.1	99.36
			3	3	187	170000	99.05
